# Seasonal variation in affective and other clinical symptoms among high-risk families for bipolar disorders in an Arctic population

**DOI:** 10.3402/ijch.v74.29671

**Published:** 2015-11-19

**Authors:** Sami Pirkola, Heidi A. Eriksen, Timo Partonen, Tuula Kieseppä, Juha Veijola, Erika Jääskeläinen, Eeva-Maija Mylläri-Figuerola, Paula M. Salo, Tiina Paunio

**Affiliations:** 1 Department of Psychiatry, University and University Hospital of Oulu, Oulu, Finland; 2 National Institute of Health and Welfare, Helsinki, Finland; 3 Depertment of Social Psychiatry, School of Health Sciences, University of Tampere, Tampere, Finland; 4 Centre of Arctic Medicine, Thule Institute, University of Oulu, Oulu, Finland; 5 Helsinki University Central Hospital, University of Helsinki, Helsinki, Finland; 6 Lapland Hospital District, Finland

**Keywords:** bipolar disorder, seasonal variation, endophenotype, mood, distress, high risk family

## Abstract

**Background:**

In bipolar disorder (BD), seasonality of symptoms is common and disturbances in circadian rhythms have been reported.

**Objectives:**

We identified high-penetrance families in a geographically restricted area in Northern Fennoscandia and studied the seasonal variation of clinical symptoms among BD subjects and their healthy relatives.

**Design:**

We explored the clinical characteristics of subjects living in Northern Fennoscandia, with extreme annual variation in daylight. Among known indigenous high-risk families for BD, we compared the affected ones (N=16) with their healthy relatives (N=15), and also included 18 healthy non-related controls from the same geographical area. Seasonal fluctuation in clinical measures was followed up at the 4 most demarcated photoperiodic time points of the annual cycle: around the summer solstice and autumn equinox in 2013, the winter solstice in 2013/2014, and the spring equinox in 2014. In the baseline, lifetime manic symptoms [Mood Disorder Questionnaire (MDQ)] and morningness–eveningness questionnaire type (MEQ) were registered, whereas in the follow-up, depressive [Beck Depression Inventory (BDI)] and distress [General Health Questionnaire (GHQ-12)] symptoms and alcohol consumption and sleep were recorded.

**Results:**

Possibly indicative or statistically significant differences in symptoms between the affected subjects and their healthy relatives were the BDI winter (13.3 vs. 2.6, t=−2.51, p=0.022) and spring scores (12.6 vs. 3.2, t=−1.97, p=0.063) and GHQ winter (4.2 vs. 0.82, t=−2.08, p=0.052) and spring scores (3.8 vs. 0.82, t=−1.97, p=0.063). Scores were higher among the affected subjects, exceeding a possibly diagnostic threshold (10 and 3) at all the time points, and without the notable seasonality which was observed among the healthy relatives. In the overall population, MDQ and MEQ scores had an inverse correlation (−0.384, significant at 0.016), indicating increased lifetime manic behaviour among “the night owl” chronotype subjects.

**Conclusions:**

In an Arctic population sample, we found different seasonal fluctuation in mood and distress symptoms and sleep duration scores between subjects with bipolar spectrum disorders and their healthy relatives. Despite the relatively small sample size, the results indicate that the symptoms and signs of BD relate to a disturbance in seasonal variation. Seasonal variation can be considered as an interesting endophenotype for BD and a promising target for further genetic studies.

Bipolar disorder (BD) is a recurrent, episodic mood disorder, characterized by alternating periods of manic and depressive behaviour (1,2). Additional subtypes of BD have been described, and certain other psychiatric disorders have bipolarity of mood among their core symptoms (3). These include cyclothymia and schizoaffective disorder, which are considered to belong to a wider spectrum of BDs (4,5).

According to population studies, the lifetime prevalence of bipolar type I disorder is between 0.2 and 3.3% (6), and of the wider spectrum of BDs between 2.4 and 15.1% (7). Epidemiological studies have previously reported regional variation as well as some particularly high-penetrance families in the distribution of BD in Finland (8,9). Genetic and specific environmental factors have explained the variance in illness liability, yielding a heritability estimate range of 0.67–0.93 (10), and associations with some common risk variants have been reported (11–13).

The role of environmental factors in the risk of onset of BD remains more controversial (14,15). Stressful life-events, substance abuse and sleep cycle disturbances are known to precipitate individual mood episodes, although the causality in these associations is probably reciprocal and complex (16,17).

There is evidence for an increase in the incidence of mood episodes as a result of rapid changes in exposure to daylight, especially in northern areas (18) but also in other parts of the world (19,20). Individuals with BD have been reported to experience seasonality in symptoms (21), and up to 20–25.5% of bipolar patients may present with a seasonal pattern (22,23). Circadian and seasonal rhythms are often disturbed in BD, and changes in these rhythms can induce mania (24,25). Current data indicate an increased incidence of mania hospitalizations during the spring season in northern Finland (Svirskis et al., manuscript in preparation).

In northern parts of the world, seasonal variation in mood and behaviour appears to be convergent with activities in social and occupational life. Annual fluctuation in mood and social activity may have been adaptive in Arctic areas, with variation in available food and other resources according to seasons of the year (26,27). In addition, circadian typology regarding the sleep–wake cycle indicates a tendency towards the “morning type” being more prevalent in the north (25).

The aim of this study was to explore the seasonal variation associated with BD in northern latitudes, using affected subjects and their relatives in families with multiple cases of BD. We hypothesized that the strong annual variation in external conditions in very northern latitudes could have led to disturbed adaptation among the affected subjects, possibly representing an etiological factor in the disease process.

## Present investigation

### Subjects

We explored the clinical characteristics of 3 groups of subjects living in extreme northern lighting conditions. Group 1 consisted of subjects suffering from bipolar spectrum disorder in pedigrees with known high prevalence of these disorders; Group 2 were healthy family controls for the affected; and Group 3 were healthy non-related controls living in the same geographical area. We investigated the possible seasonal fluctuation in mood, distress, sleep, social activity and alcohol consumption of subjects in Groups 1 and 2 by monitoring these parameters during a period of 1 year at the 4 most demarcated or extreme seasonal time points, around the 2 solstices and equinoxes. Baseline measurements and their correlations were made for all 3 groups. As a specific feature of our setting, Groups 1 and 2 represented to a large extent the indigenous population of the northern latitudes, the Sámi people, who have settled the area for approximately 8,000–10,000 years.

The Sámi people are the only indigenous people in Europe. For the most part, they live in the northernmost area of Scandinavia, including parts of Finland, Norway and Sweden. Genetically, the Sámi people are a unique population (28), which differs from other Scandinavians.

**Fig. 1:**
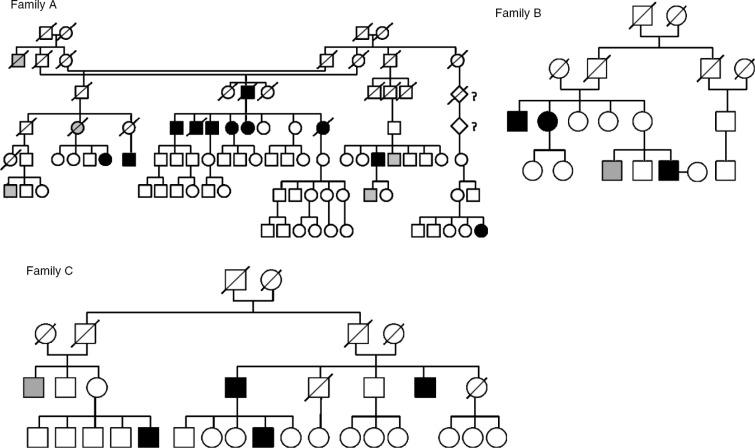
Families A, B and C with bipolar subjects (black).

During their routine clinical work at a health centre, the health-care professionals had observed a high incidence of probable bipolar subjects in 3 Sámi family branches (Families A, B and C) in northernmost Fennoscandia, between latitudes 68°N and 70°N. Family A (N=20) included a family in which 4 out of 6 siblings had known, diagnosed and lithium-treated BD type I, and bipolar illness occurred in the same pedigree with a notably high frequency. Similar observations held for another family branch (family B, N=8) as well as for a third family C (N=3) from the Sámi population (Fig. 1). In cases of lack of hospitalization and consequent clinical diagnoses, a structured clinical interview (SCID I) (29) and a set of structured questionnaires about mental health symptoms were used to obtain clinical diagnoses. Altogether, 16 subjects with a bipolar spectrum disorder were identified in the protocol. Healthy relatives (n=15) were selected to attend as family control subjects, and 18 non-related subjects living in the same area and lighting conditions were chosen as controls, particularly for further genetic studies.

### Ethical issues

The research plan was reviewed and approved in The Regional Ethics Committee of the Northern Ostrobothnia Hospital District. The participants were informed in detail about the objectives and proceedings of the study, and they were allowed to withdraw at any point. They gave a written informed consent regarding their participation. Their travel expenses and a lunch voucher for the examination day were reimbursed. As an additional benefit, the expertise of the mental health professionals was at their disposal in case of need for consultations or more general information. The study process was reviewed and considered by the authors to follow the principles of the WMA Helsinki Declaration.

### The study periods

**Fig. 2:**
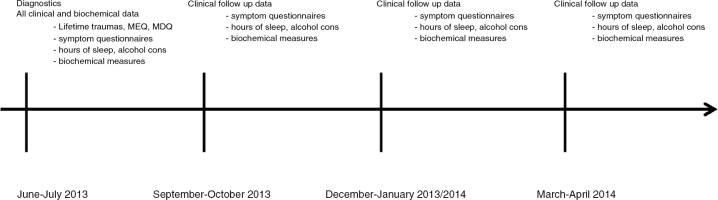
Time points of data collection.

The clinical follow-up data and blood samples were collected at Arctic latitudes during 3-week periods between July 2013 and March 2014 during the 4 most demarcated periods of light exposure of the year: the period of most light around midsummer (SUMMER, S1); the period of the fastest decrease in light exposure around September–October (FALL, S2); the darkest period around December–January (WINTER, S3); and the most rapidly increasing light period in March–April (SPRING, S4) (Fig. 2).

### Data collection

Among the subjects from Arctic high-risk families for bipolar spectrum disorders (Groups 1 and 2), the initial evaluation for psychiatric disorders and their symptoms included a clinical interview, the Structured Clinical Interview for Psychiatric Disorders (SCID I) (29), in addition to patient record data. Furthermore, we evaluated their baseline characteristics, including lifetime manic symptoms assessed with the Mood Disorder Questionnaire (MDQ) (30,31), lifetime traumatic experiences assessed with the Trauma Screening Questionnaire (TSQ) (32) and the circadian preference for daily activities assessed with the Morningness–Eveningness Questionnaire (MEQ) (33).

In a follow-up at the same 4 time points, we collected data on mood and distress as assessed with the Beck Depression Inventory (BDI) (34) and the General Health Questionnaire (GHQ-12) (35), questionnaire-based alcohol consumption (grams per week) and a sleep duration questionnaire (hours per day).

The data collection was conducted by the local chief medical practitioner, assisted by local community nurses and a psychiatric resident, of which 1 community nurse and the psychiatric resident had received formal training for SCID – interviewing.

### Statistical analyses

Due to the relatively small sample size, we also report p-values from 0.1 to 0.05 as showing a probable tendency to significance, in order not to miss any finding possibly indicative of a true difference. We used the bivariate correlation test, the t-value for individual samples and one-way ANOVA for testing the differences in clinical measures at the baseline and at the different time points, but we tested mainly the differences between affected and healthy relatives in the identified pedigrees. We used the General Linear Model test for repeated measures in testing the variance in clinical measure scores within the affected and the healthy subjects. IBM SPSS 23 software was used for the analyses (35,36).

## The results

### Characteristics of the sample

The participants from the families did not differ significantly between the affected and the healthy ones in their age or sex (mean 59.9 years vs. 50.9 years, t=−1.35, p=0.189; M/F 60% vs. 75%, Fisher's test, p=0.458). The majority of the affected (12/16) had BD type I, 1 had BD type II, 2 had cyclothymia, and 1 had schizoaffective disorder. Five of the 15 healthy relatives had some kind of depressive disorder. The healthy non-related controls had been collected via the health centre mainly for further genetic controls and baseline information, and they were not matched for age or sex: 89% of them were females and mean age was 54.8 years. They were not interviewed for clinical diagnoses.

### The baseline scores and their associations

In baseline measurement, the subjects with a bipolar spectrum disorder had the highest MDQ scores (9.0 vs. 2.5 vs. 1.0, F=23.36, p<0.01), when compared with the pedigree controls and non-related controls. Differences in the TSQ and MEQ appeared to be statistically non-significant for the 3 groups.

Correlations between the lifetime MDQ scores and several clinical measures in the overall population including the non-related controls are presented in Table I. The MDQ and MEQ sum scores had an inverse correlation (r=0.384, P=0.016), and the MDQ sum score correlated positively with BDI and GHQ sum scores at the follow-up.

**Table I:** Correlations of baseline measurements with the follow-up symptom data

		MEQ sum	TSQ sum	MDQ sum	S1 BDI sum	S1 GHQ sum	S2 BDI sum	S2 GHQ sum	S3 BDI sum	S3 GHQ sum	S4 BDI sum	S4 GHQ sum
MEQ sum	Pearson correlation	1	−0.228	−0.384*	−0.113	−0.117	0.186	0.011	−0.122	−0.092	−0.069	−0.055
	Sig. (2-tailed)		0.181	**0.016**	0.553	0.537	0.362	0.96	0.57	0.671	0.726	0.784
	N	42	36	39	30	30	26	24	24	24	28	27
TSQ sum	Pearson correlation		1	0.323	0.667**	0.282	−0.154	0.033	0.268	−0.139	0.061	−0.068
	Sig. (2-tailed)			0.055	**0.001**	0.155	0.493	0.889	0.228	0.538	0.767	0.747
	N	36	36	36	27	27	22	20	22	22	26	25
MDQ sum	Pearson correlation			1	0.515**	0.448*	0.431*	0.332	0.498*	0.466*	0.381*	0.382
	Sig. (2-tailed)				**0.006**	**0.019**	**0.04**	0.142	**0.016**	**0.025**	**0.05**	0.054
	N	39	36	39	27	27	23	21	23	23	27	26

Values in bold represent the significant p values. MEQ: Morningness–Eveningness Questionnaire; TSQ: Trauma Screening Questionnaire; MDQ: Mood Disorder Questionnaire; BDI: Beck Depression Inventory; GHQ: General Health Questionnaire.

* Correlation is significant at the 0.05 level (2-tailed).

** Correlation is significant at the 0.01 level (2-tailed).

### Seasonal variation in symptoms among affected subjects and their healthy relatives

**Fig. 3:**
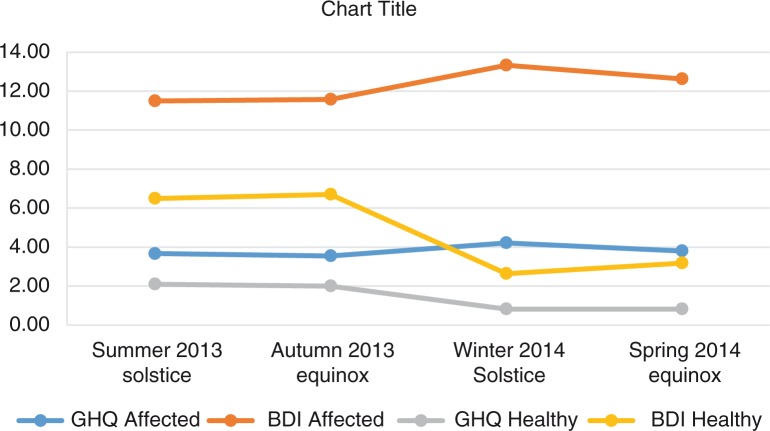
GHQ-12 and BDI symptom scores during the follow-up.

At the follow-up, we measured the symptoms of depression (BDI) and distress (GHQ-12), sleep and alcohol use between the subjects with a bipolar history and the non-affected controls during 4 time points of the year. The results are presented in Table II and Figure 3.

**Table II:** Clinical variable scores in the follow-up points during the year

Group	Measures	Summer 2013 Mean (SD)	Autumn 2013 Mean (SD)	Winter 2014 Mean (SD)	Spring 2014 Mean (SD)
Affected subjects (N=16; M12, F 4)	GHQ-12	3.67 (5.21)	3.55 (5.07)	4.22* (4.76)	3.80* (4.85)
*MDQ*: 8.96 (SD 4.06)**	BDI	11.50 (11.67)	11.58 (11.04)	13.33** (13.78)	12.64 (15.47)
*TSQ*: 1.75 (SD 3.11)	Alcohol consumption	161.17 (393.10)	474.35* (597.45)	168.61 (377.75)	343.59 (816.63)
*MEQ*: 49.20 (SD 4.13)	Sleep hours	8.67 (2.64)	9.29 (2.14)	8.44* (1.69)	9.18** (1.89)
Healthy family controls (N=15; M 9, F 6)	GHQ-12	2.10 (3.03)	2.00 (2.36)	0.82* (1.25)	0.82* (1.25)
*MDQ*: 2.49 (SD 3.21)**	BDI	6.50 (6.20)	6.70 (5.48)	2.64** (3.07)	3.18 (3.17)
*TSQ*: 0.67 (SD 1.61)	Alcohol consumption	50.88 (61.74)	72.81* (88.98)	45.99 (62.72)	58.70 (70.12)
*MEQ*: 51.21 (SD 3.60)	Sleep hours	8.25 (1.09)	7.32** (1.06)	7.41* (0.92)	7.60** (1.02)

MEQ: Morningness–Eveningness Questionnaire; TSQ: Trauma Screening Questionnaire; MDQ: Mood Disorder Questionnaire; BDI: Beck Depression Inventory; GHQ-12: General Health Questionnaire 12 items.

* p<0.01, between affected and healthy relatives (one-way ANOVA).

** p<0.05, between affected and healthy relatives (one-way ANOVA).

Possibly indicative or statistically significant differences in symptoms between the affected and the healthy relatives were the BDI winter (13.3 vs. 2.6, t=−2.51, p=0.022, Cohen's d=1.0712, η^2^=0.260) and spring scores (12.6 vs. 3.2, t=−1.97, p=0.063; Cohen's d=0.8403, η^2^=0.163) and GHQ winter (4.2 vs. 0.82, t=−2.08, p=0.052, Cohen's d=0.9024, η^2^=0.193) and spring scores (3.8 vs. 0.82, t=−1.97, p=0.63, Cohen's d=0.8421, η^2^=0.170). The affected had significantly more sleep in the fall (9.3 vs. 7.3 h, t=−2.61, p=0.017, Cohen's d=1.1522, η^2^=0.232), and also in the spring. The affected had higher BDI and GHQ scores at all times, exceeding the established threshold values for possible depressive or anxious states (BDI ten points and GHQ-12 three points) (35). The BDI scores for the healthy relatives, but not for the affected individuals, varied between the 4 time points (GLM: F=22.210, significant at 0.043 vs. F=1.919, significant at 0.303).

## Discussion

In an Arctic population sample, we found different seasonal fluctuation in mood and distress symptoms and sleep duration scores between subjects with a bipolar spectrum disorder and their healthy relatives. Although the study sample is relatively small, the results suggest that the manifestation of BD relates to a disturbance in seasonal variation. It appears that, in families with multiple cases of BDs and putatively with a high genetic risk, those with the disorder have a distinct, possibly disturbed pattern of seasonal fluctuation. The affected may have lost a natural, season-syntonic variation in their general activity, and they may be prone to a relative elevation of depressive and distress symptoms during spring- and summertime, with more daylight.

A marked difference between the affected individuals and their relatives in the seasonal assessments of depressive and distress symptoms and sleep duration was observed from time point to time point during the follow-up. A relative winter and spring elevation of depressive and distress symptoms among the affected is convergent with the reported winter and spring activation of bipolar spectrum disorders. In the families with multiple affected individuals, those with the disorder phenotype expressed continuing affective and distress symptoms from winter to spring, whereas the symptom level of the non-affected siblings decreased. Furthermore, these fluctuations in mood and sleep were preceded by significant differences in the level of alcohol intake in the autumn.

One finding deserves a closer look. First, the higher the MDQ score was, the lower the MEQ score was. This indicates that the closer to the “night owl” a subject's chronotype was, the more likely were lifetime manic symptoms. Our finding is original, and it agrees with earlier reports that among “night owls” the probability of either type 1 or type 2 BDs is increased (37,38), and that during a follow-up of 4 years on average, patients with BD remain as “night owls” regardless of their prevailing mood state (39). This is particularly interesting in the context of a general increased prevalence of the morning type in northern latitudes (25).

In general, the period from the winter solstice to the spring equinox includes a gradual increase both in day length and in social activation, with increasing mobility. The lack of fluctuation in clinical symptoms could be seen as a season dystonic pattern, and as a failure to adapt to increase in external light and social activation. The biological mechanisms will be an interesting focus for future studies including analysis of variations in genetic regulatory mechanisms during different seasons.

Some limitations should be noted. Due to the sparse population in the study area, the sample in this work was relatively small. The setting was built on observations of certain pedigrees having a surprisingly high incidence of bipolar spectrum disorders, and a hypothesis on the seasonality of the phenotype was then to be tested by clinical measures. Having only 16 vs. 15 subjects in the pedigrees decreases the statistical significance of the findings and leaves a possibility for both type 1 and type 2 statistical errors.

The third group, consisting of healthy non-related subjects, were not age- or sex-matched with Groups 1 and 2, but rather they were mainly females. Therefore, their clinical follow-up measures were incomparable with the family subjects, and they were only used for validation of the baseline measures (Table I).

As a strength, the attrition rate was high, and at least 1 DNA sample and baseline clinical data were obtained from all the identified bipolar subjects. This most probably indicates the community health centre team's good motivational skills and understanding of the local conditions.

## Conclusions

According to our small sample study, the symptoms and signs of BD in Arctic latitudes relate to a disturbance in seasonal variation. Seasonal variation is an interesting intermediate phenotype for BD and a promising target for further genetic studies.
